# Supplementary material for: Nonlinear wavefront shaping with optically induced three-dimensional nonlinear photonic crystals
Source: Nat Commun. 2019 Jul 19;10:3208. doi: 10.1038/s41467-019-11114-y (PMC6642115; doi:10.1038/s41467-019-11114-y)
Supplement: Supplementary file 1 — Supplementary information [file 41467_2019_11114_MOESM1_ESM.pdf]

Supplementary Information for "Nonlinear wavefront shaping with optically-induced three-dimensional nonlinear photonic crystals"

Liu et al.

Supplementary Note 1: Characterization of laser-induced ferroelectric domain structures with piezo-response force microscopy (PFM)

We used PFM[1] to confirm that the structures formed by femtosecond laser writing are formed by realignment of ferroelectric domains and not material modifications such as optical damage. To this end we fabricated a structure of rectangular array of 3 micrometer wide circular domains which was located at and just below the surface of the crystal. The ferroelectric domains were measured using a resonant-enhanced PFM (MFP-3D, Asylum Research). Pt/Ir coated conductive AFM tip SCM-PIT-V2 (resonance frequency 75 kHz, spring constant 3 N/m, radius ~25 nm) were used to obtain the PFM results. Out-of-plane polarization is measured by detecting the tip-deflection signal at the resonant frequency of modulation. The antiparallel domains with out-of-plane polarization can be seen in the PFM phase image (Fig. Supplementary Figure 1b). It is clearly seen the uniform domain orientation in the laser-processed regions. Since the host crystal contains random distribution of the oppositely oriented sub-micron size domains, the phase profile exhibits random oscillations in the regions surrounding the fabricated structures. Meanwhile, the AFM topography (Supplementary Figure 1a) shows there is no optical damage or defects caused by the illumination of the writing laser beam.

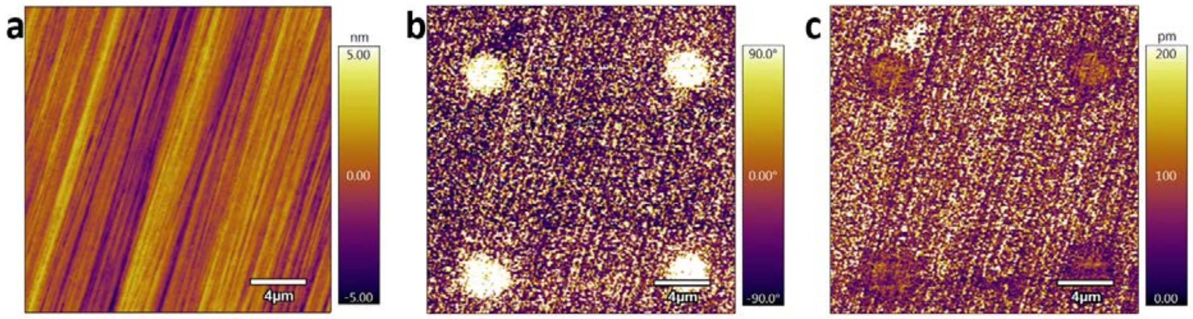

Supplementary Figure 1 Characterization of laser-induced ferroelectric domain structures. The AFM topography (a), PFM phase (b), and amplitude (c) images obtained in a laser-written $Ca_{0.28}Ba_{0.72}Nb_2O_6$ (CBN) crystal. The laser written area has a form of a square array of circular spots (60x60 microns). Only its representative fragment with four spots is shown here.

Supplementary Note 2: Determination of the topological charge of the second harmonic vortex beam by using astigmatic transformation approach

We confirmed the second harmonic topological charge by using astigmatic transferring the optical vortex beam through a cylindrical lens[2-4]. Supplementary Figure 2 illustrates this approach. The resulting intensity distribution in the focal plane of the cylindrical lens exhibits two tilted parallel dark stripes indicating the charge l_{SH}=2.

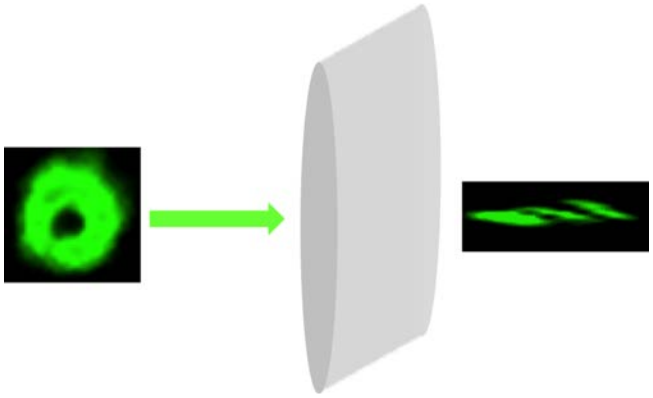

Supplementary Figure 2 Illustrating diagnostics of the ultrafast vortex pulses. The input vortex beam passes through a cylindrical lens undergoing an astigmatic transformation. The resulting light intensity distribution observed in the focal plane of the lens consists of tilted dark lines on an elongated bright background. The number of the dark lines equals to the topological charge of the vortex. Here the two dark lines indicate the charge l_{SH}=2.

Supplementary References:

1. Gruverman, A. & Kalinin, S. V. Piezoresponse force microscopy and recent advances in nanoscale studies of ferroelectrics. *J. Mater. Sci.* **41**, 107 (2006).
2. Denisenko, V., Shvedov, V., Desyatnikov, A., Neshev, D., Krolikowski, W., Volyar, Soskin, A., M. & Kivshar, Yu. Determination of topological charges of polychromatic optical vortices. *Opt. Express* **17**, 23374 (2009).
3. Shvedov, V. G. , Hnatovsky, C., Krolikowski, W. & Rode, A. V., Efficient beam converter for the generation of high-power femtosecond vortices, *Opt. Lett.* **35**, 2660-2662 (2010).
4. Alperin, S.N., Niederriter, R. D., Gopinath, J. T. & Mark E. Siemens, Quantitative measurement of the orbital angular momentum of light with a single, stationary lens. *Opt. Lett.* **41**, 5019-5022 (2016).
